# Sleep Disorders in Early Childhood and the Development of Mental Health Problems in Adolescents: A Systematic Review of Longitudinal and Prospective Studies

**DOI:** 10.3390/ijerph182211782

**Published:** 2021-11-10

**Authors:** Lawrence T Lam, Mary K Lam

**Affiliations:** 1 Tung Wah College, Hong Kong, China; 2 Faculty of Health, University of Technology Sydney, Sydney, NSW 2007, Australia; 3 RMIT University, Melbourne, VIC 3083, Australia; mary.lam@rmit.edu.au

**Keywords:** early childhood, sleep disorders, mental health problems, adolescents, longitudinal studies, systematic review

## Abstract

The association between sleep problems, particularly sleep disorders, and mental health has long been studied and recognized. However, the causal relationship between sleep disorders, particularly during early childhood, on mental health problems in adolescence are yet to be established. From a preventive perspective, it is important to understand the causality of mental health problems in adolescents so that intervention measures can be derived and implemented as early as possible for maximum effectiveness. To provide more precise information on the effect of early childhood sleep disorders on mental health problems during adolescence, a systematic review was conducted on longitudinal and prospective studies reported in the literature. Following the PRISMA guidelines with an extensive search of the literature 26 studies were identified. Seven of these identified studies satisfied all selection criteria with sufficient data on the effect of early childhood sleep disorders and mental health problems in adolescence. Information was extracted and analyzed systematically from each study and tabulated. The overall results obtained from these studies indicate a significant and possible causal relationship between early childhood sleep disorders and the development of mental health problems, such as anxiety, depression, and ADHD in adolescence. These results are discussed with regards to the theoretical and practical implications as well as preventive strategies.

## 1. Introduction

The association between sleep problems, particularly sleep disorders, and mental health has long been established [1,2,3,4]. From the epidemiological perspective, this association could either be unidirectional or bidirectional in terms of the relationship between these two health conditions [5,6,7]. Two possible scenarios could result in a unidirectional associative relationship between these two conditions. First, among others, sleep disorders are one of the risk factors in the causal pathway of mental health problems. In this case, sleep disorders precede mental health problems in the developmental trajectory of the latter [8]. Second, mental health problems and sleep disorders are comorbidities, with sleep disorders a manifestation of underlying mental health problems [9]. For the bidirectional understanding of the association between these two conditions, there could be a mutual and reciprocal cause-and-effect relationship operating between each, such that mental health problems may induce sleep disorders and, in return, sleep disorders exert an effect on mental health problems at different phases of development. This has been demonstrated in the relationship between mental health and other health conditions [10]. 

It has been well-established that adolescence is a period when young people’s mental health can be greatly affected by many personal and environmental risk factors [11]. The developmental trajectory of mental health problems is complex and could span many years [12]. From a preventive perspective, it is important to understand the causality of mental health problems in adolescents so that intervention measures can be derived and implemented as early as possible for maximum effectiveness [13]. To determine the causal pathway of mental health problems in adolescents, it is important to establish the temporality of the event sequence and to gain as much information as possible on various aspects of young people’s growth through different stages from infancy, early childhood, to young adolescence [13]. Hence, in the search for sleep disorders as a causal factor of mental health problems, a longitudinal and prospective approach to information gathering following a cohort of young children from a very early stage of development to adolescence would be ideal [13]. This approach could be powerful in providing data for testing the hypothesis that childhood sleep disorders, particularly in infancy and early childhood, contribute to and may also be causative of mental health problems at the later stage of life. This could also be considered as evidence for the establishment of the causal relationship between early childhood sleep disorders and adolescent mental health problems. 

Contrasting definitions of childhood exist. A child is defined by the United Nations in the “Convention on the Rights of the Child” as a person with an age of 18 years or younger [14], however, the World Health Organization (WHO) defines adolescents as people between 10 and 19 years of age [15]. Therefore, a large proportion of adolescents are also considered as children by these two definitions. However, for early childhood, a clearer definition has been found—the WHO defines early childhood as the period between 0–8 years [16]. To maintain the consistency of terms and definitions, the WHO definitions of early childhood and adolescence were adopted. In terms of sleep disorders in children, the definition provided by the American Academy of Sleep Medicine (AASM) was used [17]. Some common sleep disorders have been identified and included as childhood sleep disorders, such as obstructive sleep apnea, parasomnias, behavioral insomnia, delayed sleep phase disorder, and restless legs syndrome [18]. For adolescent mental health problems, the standard definition and diagnostic criteria of the Diagnostic and Statistical Manual of Mental Disorders – 5th Edition (DSM-5) by the American Psychiatric Association (APA) were followed [19]. Common adolescent mental health problems include, but are not limited to: anxiety disorder, mood disorders, attention deficit and hyperactivity disorders, and disruptive behavior disorders [19]. 

To ascertain any similar and relevant existing systematic reviews on the same topic, a search of the main health-related databases had been conducted prior to the commencement of the review study. The results suggested no systematic review on the same topic has been reported in the literature. To bridge this gap of knowledge, this review study aims to examine the effect of sleep disorders during early childhood, as defined above, on the development of various mental health problems in adolescents.

## 2. Methods

Following the PRISMA guidelines for systematic reviews, a structured and systematic approach for the literature search and review process was conducted [20]. For the literature search, major medical and health, as well as psychological and developmental databases, were used. These included (1) PubMed, (2) PsyInfo, (3) Web of Science, (4) SCOPUS, (5) CINAHL, and (6) ERIC.

For the keywords search, the syntax used were: (“sleep problems AND children”) AND (mental health problems AND adolescents) AND (longitudinal studies OR prospective studies). Slight modifications to the syntax entry were allowed based on the database requirements. Some restrictions were imposed on the search, including that they must be peer-reviewed journal articles written in the English language and be original studies. There was no restriction on the date of publication. 

The online application “Mendeley Reference Manager” was employed to collect the titles, abstracts, and full articles retrieved from the initial literature search. A database housing all titles from the initial search was created. The following steps were followed to ensure all selection criteria were met by the selected studies for final data extraction. First, abstracts were screened and analyzed for the study types with sleep problems in early childhood as the study/exposure variables and mental health problems in adolescents as the outcome. Second, full texts of the potentially acceptable articles were examined to ensure that they were suitable for final data extraction. This step was conducted by both authors (L.L. and M.L.) independently based on the selection criteria. At the end of the independent selection, the results of selection by the authors were matched for any discrepancies. Differences on selection were discussed and discrepancies were resolved by drawing references to the selection criteria. Finally, reference lists for the selected articles for data extraction were also examined to further ensure that no other relevant studies were missed during the literature search.

The following criteria were applied for the selection of studies: (1) the study type of the publications must be a longitudinal design with the study and outcome variables identified according to the definition above; (2) clear descriptions of study methods must be provided, allowing an accurate assessment on whether sleep problems were examined during early childhood as defined with a follow-up assessment for mental health problems at adolescence; (3) studies that included the tools and procedure for sleep problems and follow-up mental health assessments; (4) studies that provided clear results allowing for an estimate of the effect of sleep problems on mental health; and (5) studies published in the English language. Longitudinal and prospective studies with assessments of both sleep and mental health problems conducted during early childhood or adolescence, as defined above, were excluded from this review.

**Figure 1:**
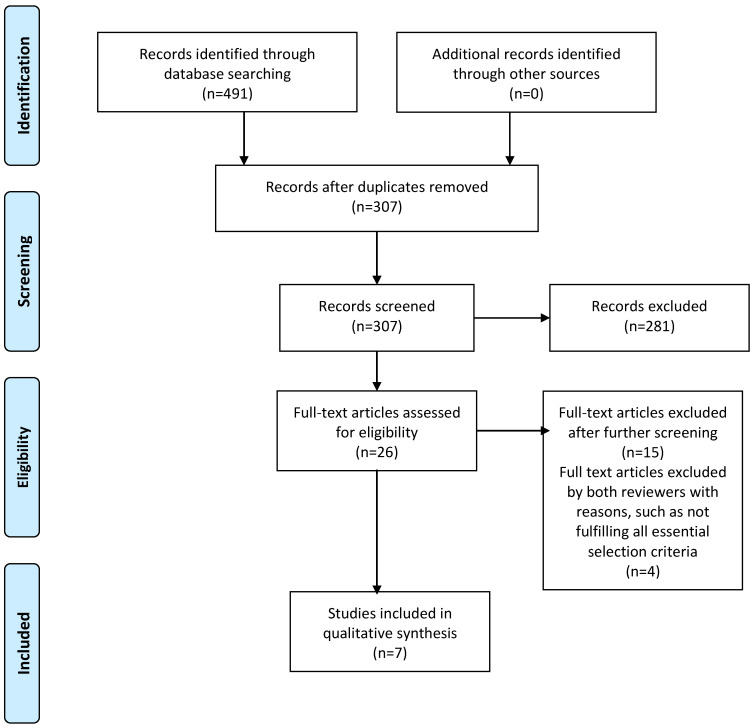
PRISMA flowchart of the search for peer-reviewed journal articles.

Upon finalizing the list of selected studies, data extraction was then conducted. Systematic extractions of information on study location, description of study design, and information on the demographic characteristics of the sample were carried out. Moreover, the tools or instruments used to assess sleep and mental health problems were also identified and recorded, and the results on the association between early childhood sleep problems and adolescent mental health problems were summarized. Comments on any potential biases and/or limitations of the studies were also presented with a descriptive approach. The data extraction and content analyses followed a standard procedure including the following steps: (1) a template for data extraction was designed as a table for information display; (2) a search for information on each of the aforementioned domains was conducted and the relevant data were recorded; (3) upon completion of the data extraction, the full study was then reviewed again for its strength and limitations. The information pertaining to the potential causal relationship between sleep problems and the subsequent mental health in adolescence was further summarized in a table. The standard PRISMA chart was used to depict the systematic literature searches and review process schematically [20] (Figure 1).

## 3. Results

The procedures described in the methods were applied for an extensive search on the six electronic databases. Twenty-six articles on the relationship between sleep problems at an earlier stage of life and subsequent mental health problems were identified. Of these, 11 were selected as potential studies for further analyses to be reviewed by both authors with an examination of the full text [21,22,23,24,25,26,27,28,29,30,31]. After concordance discussions and resolution of any discrepancies, it was found that seven articles satisfied the selection criteria fully with statistical information on the relationship between early childhood sleep disorders and mental health problems in adolescence [22,25,26,27,28,29,30]. The main reasons for the four articles to be excluded were: (1) the focus of the study was not on early childhood sleep disorders, but sleep problems during adolescence and the association with a mental health problem at a later stage of life development; (2) the study did not provide sufficient information for the effect estimate, or (3) the assessment of the exposure and/or the outcome variables did not fulfill the selection criteria [21,23,24,31]. Detailed information was extracted from these seven articles and is summarized in Table 1.

In terms of the study design, nearly all utilized a standardized self-reported instrument or a set of questions on sleep problems to collect baseline data on early childhood sleep disorders. Two studies employed the Sleep Problem Scale of the Child Behavior Checklist (CBCL), two used the Sleep Habits Questionnaires (SHQs), and another two studies applied a set of questions on sleep problems (Table 1). One of the studies using SHQs also used unattended home polysomnograms to assess early childhood sleep apnea [25]. Slykerman’s study, mainly focusing on the duration of sleep at a later stage of early childhood, employed actigraphy to measure the exposure variable [30]. For the outcome variable, namely adolescent mental health problems, most of the studies focused on anxiety, depression, and attentional problems such as attention deficit and hyperactivity disorder (ADHD). Two studies also included externalizing behaviors as the outcomes [26,27]. One study investigated the effect of early childhood sleep problems on symptoms of borderline personality disorder (BPD). At the same time, another concentrated on psychotic symptoms in adolescents while using the same data set for their secondary data analysis studies [28,29]. Slykerman’s study adopted a more generic outcome of emotional/behavioral problems in early adolescents as the outcome variable while also examining ADHD [30]. In terms of the measuring instruments for mental health problems, an array of different validated instruments was reported. While most of the studies employed the CBCL or the youth version, the Youth Self Report (YSR) and other instruments were also utilized as the assessment tool used for adolescent mental health. These included the MacArthur Health and Behavior Questionnaire (HBQ) [26], the UK Childhood Interview for DSM-IV-Borderline Personality Disorder [28], Psychotic-Like Symptom Interview [29], and Strengths and Difficulties Questionnaire (SDQ) [30]. 

For the follow-up period, the cohorts engaged in these studies ranged from 4 to 14 years. All these were large-scale studies with sample sizes ranging from 304 to 7155. However, two secondary data analysis studies with large sample sizes utilized data collected from the same longitudinal cohort with a similar follow-up period suggesting duplication of samples [28,29]. Hence, after adjusting for the possible duplications, a total sample of about 10,000 children was included in these studies. 

Review results are summarized in Table 2. As indicated, there was some evidence for a predictive relationship between early childhood sleep problems, particularly common symptoms of sleep problems such as insomnia, nightmares, trouble falling asleep and short sleep duration, and ADHD in adolescents. Of the three studies focusing on this adolescent mental health problem, all yielded positive and significant results, although different analytical approaches were employed (Table 1). For example, Gregory and O’Connor found that sleep problems at age 4 significantly predicted attentional problems during mid-adolescence (β = 0.11, *p* < 0.05, R2 = 0.25) [22]. For adolescent anxiety and depression, common sleep problems and persistent insomnia were found as significant predictors in two studies (Gregory and O’Connor, 2002; Armstrong et al., 2013) [22,26]. Silva et al. also found that a short duration of sleep (<7.5 h/night) in childhood was indicatively predictive of anxiety/depression during adolescence, however, the relationship was not significant at 5% Type I error rate (OR = 3.3, 95% CI = 0.83–13.5) [25]. In terms of other mental health problems, the evidence for early childhood sleep problems as a potential predictor was not sufficiently strong due to the small number of studies. Regarding the quality of these studies, the majority were well-conducted or used data collected from well-executed and large-scale studies with appropriate analytical approaches. A common shortcoming in most of these studies was that assessments on the exposure and outcome variables were mainly by self-reporting from parents and/or by the child, with only two utilizing objective measures on sleep problems. This might constitute a risk on reporting basis, although it would be non-systemic and non-differential.

## 4. Discussions and Conclusions

This study aims to investigate the possible causal relationship between early childhood sleep problems and subsequent mental health problems in adolescence through systematically reviewing the evidence provided by studies of strong design. It is hypothesized that early childhood sleep problems, as defined in the previous section, are detrimental to the development of the child and are possibly a causal factor of mental health problems during adolescence. The results obtained from this review suggest that common sleep problems and persistent insomnia in early childhood are significantly related to ADHD and are predictive of symptoms of anxiety and depression in adolescence. Since there has not been any similar systematic review study conducted and reported in the literature, comparison of results would not be possible. Hence, the results obtained from this review study would be considered unique. The longitudinal and prospective design of the included studies for review could provide evidence of a higher level in terms of the evidence-based model [32]. Hence, a greater confidence in the conclusion that the association between early childhood sleep problems and mental health problems, particularly attentional problems, anxiety, and depression, in adolescence could be in a causal relationship [33]. However, due to some intrinsic issues with the design of these studies that will be further explored later in this section, these results would not be able to provide an answer for the question of whether such a relationship is unidirectional or bidirectional. This is worthy of further exploration. For other mental health problems, due to the small number of studies, the strength of evidence is not ascertained with sufficient confidence. 

The results obtained from this review provided epidemiological evidence of a possible causal and predictive relationship between early childhood sleep problems and adolescent mental health problems. These results have also raised an important question as to why young children who suffer from common sleep problems would have a higher risk of developing mental health problems at a later stage of development. The mechanism and the causal pathway would be complex and may differ depending on the mental health problem. Biologically, it has long been recognized that sleep is regulated by the hypothalamus of the brain and a few neurotransmitter systems involved in sleep regulation [34,35]. These include the monoaminergic, cholinergic, and serotonergic systems, as well as the inhibitor GABAergic mechanisms that promote wakefulness. The activation of the serotonergic system also induces excitatory activity in the forebrain and plays a role in wakefulness [36]. Some of these systems, such as the GABAnergic system, have been identified as being closely related to anxiety and stress, such that dysfunction of these systems is implicated in anxiety disorders [37]. Whereas a reduction of the serotonergic function has long been found to be related to the pathogenesis of major depressive disorders [38]. Hence, disruption to these neurotransmitter systems during early childhood would have an effect on the sleep regulatory ability of the young child and impact directly on the stress response system as well as the serotonergic function of the young brain. This, if unnoticed and unattended early enough, would further compromise the stress response and mood regulatory systems paving the way for anxiety and depression development, possibly through a self-reinforcing and looping mechanism. In terms of the relationship between early childhood sleep problems and ADHD, while the underlying neurobiological mechanisms are still being investigated, Wajszilber and colleagues suggested different possible mechanisms for different sleep problems [39]. The association between sleep breathing disorders, such as sleep apneas and attentional problems, may be due to the effects of hypoxia on the brain resulting in stress or even inflammation in the brain. This, in turn, could alter the neurochemistry of the brain, particularly the prefrontal cortex, leading to neurobehavioral problems and exhibiting symptoms of ADHD. On the other hand, the linkage between persistent sleep movement and ADHD may lie in the deficit of dopamine in the nigrostriatal brain region, which has been associated with both disorders [39]. 

The indicative results obtained from this study have some important practical implications, particularly in terms of early prevention and intervention of adolescent mental health problems. The possible causal relationship between early childhood sleep problems and the development of mental health problems at a later life stage provides a possible inroad to the development of early prevention and intervention strategies for youth mental health problems. The early identification and treatment of childhood sleep problems could be one effective means of dampening the developmental trajectory of mental health problems by intervening in basic neurobiological mechanisms at very early stages of life. The view that sleep intervention at an early stage might be a preventive strategy for the onset of clinical disorders has been advocated recently by some psychiatric researchers [40]. Results from a recent randomized controlled trial (RCT) on behavioral interventions for sleep problems in young children, including parental education on the child’s sleep, the establishment of pre-sleep routines, extinction, and positive reinforcement, have demonstrated the efficacy in improving sleep outcomes [41]. It would be a prudent strategy for clinicians and parents to have a better understanding of the importance of sleep in early childhood and the relationship between sleep problems and subsequent mental health problems. 

As in all studies, there are strengths and limitations in this systematic review. This review study followed the PRISMA guidelines for systematic reviews, closely ensuring the scientific rigor of a systematic review. Reviewers also keenly observed the selection criteria for the inclusion of studies in the review, reaffirming that the conclusion drawn is based upon results with sufficiently strong evidence and with confidence. In terms of limitations pertaining to individual studies, comments were made in the information extraction table and would not be repeated here. (Table 1) However, some general limitations have been identified. First, due to the small number of studies available for the review, only a few mental health problems, mainly anxiety, depression, and ADHD, could be examined, although these are the most common adolescent mental health issues. Second, different analytical approaches have been used for investigating the effect of childhood sleep problems and adolescent mental health and for the calculation of the effect estimate. This has posed a great difficulty for pooling the statistical information together from various studies on the same outcome variables. Hence, there are insufficient data for a meta-analysis to be conducted. Third, it was observed that these studies were mainly conducted in developed countries involving Caucasian children with very few in a non-Western cultural background. Hence, the results might not be totally applicable to all children. Fourth, in most of these studies, there seemed a lack of consideration in the importance of the parental history of mental health problems as a confounding factor in the relationship of early childhood sleep problems and mental health problems in young people. 

In conclusion, early childhood sleep problems could be one of the causal factors and are part of the causal pathway of adolescent mental health problems, particularly for anxiety, depression, and ADHD. A greater understanding of early childhood sleep problems and ways to improve the sleep quality of young children should be enhanced. Tackling the sleep problems in early childhood could be an early prevention and intervention strategy for youth mental health problems.

## Figures and Tables

**Table 1:** Information extracted from the selected studies on early childhood sleep problems and mental health problems in adolescents.

Reference (Author, Year, Place)	Participants	Study Methodology	Exposure, Confounding Variables and Measures	Outcome Variable and Measures	Method of Analysis and Variables Adjusted	Results	Comments
Gregory and O’Connor, (2002), CO, USA	Participants of the study were part of the Colorado Adoption Project with children and parents recruited from adoptive and non-adoptive families. In total, 490 families were recruited with an equal number of family types.	Parents and children were recruited and followed since birth. Assessment on children was conducted annually since recruitment. Baseline assessment on sleep problems was conducted at the time when the child was 4 years old when sleep problems were first assessed. Mental health assessment was evaluated annually until 15 years old (n = 490).	**Exposure:** Early Childhood sleep problems with common symptoms of sleep disorders, including nightmares, sleepwalking and talking, trouble in falling asleep. **Measures:** The Sleep Problem Scale of the Child Behavior Checklist (CBCL) **Confounding variables:** Child sex, adoption status, anxiety/depression at age 4, attention problems at age 4, and aggression at age 4, assessed by CBCL.	**Outcome:** Anxiety/depression and attention problems. **Measures:** The Behavioral/Emotional Scale of the CBCL.A combined assessment across 13, 14, and 15 years because of attrition.	Relations between early childhood sleep problems and anxiety/depression, as well as attentional problems were examined with hierarchical linear regression with adjustment for child’s sex, adoption status, and behavioral/emotional problems at age 4.	Regression analyses results suggested sleep problems at 4 year significantly predicted anxiety/depression in mid-adolescence (β = 0.16, *p* < 0.01, R^2^ = 0.12). Similarly sleep problems at age 4 also significantly predicted attentional problems at mid-adolescence (β = 0.11, *p* < 0.05, R^2^ = 0.25).	**Strengths and Limitations:** The study was limited by a high attrition rate (26%), although the original sample was of a reasonable size (n = 490). Furthermore, the assessments on sleep problems and mental health problems were self-parents. Without an additional informant, especially the child at the adolescence period, report basis might be an issue. Some important confounding variables, such as parental mental health, might be missing
Silva et al. (2011), Tucson, USA	Children, aged 6–12 years of Hispanic and Caucasian origin were recruited in the Tucson Children’s Assessment of Sleep Apnea Study. Children were recruited from the Tucson Unified School District, excluding those who had a history of tonsillectomy and mental disorders. The mean age was 8.9 years at baseline assessment.	Children were followed after baseline assessments (n = 503) on sleep apnea and behavioral/emotional problems. They were followed for about 5 years with 304 participants retained in the study for outcome assessments. All assessments were conducted during the in-home visit (n = 304).	**Exposure:** Sleep apnea and sleep history characteristics. **Measures:** Sleep apnea was assessed using unattended home polysomnograms (PSGs). Sleep scores were calculated by a somnographic technologist who was blind to the child’s mental status. Sleep history and characteristics were assessed using the Sleep Habits Questionnaires (SHQs) by parental reports. **Confounding variables:** Baseline BMI, ethnicity, sleep apnea at follow-up, age at follow-up, caffeine use at follow-up, and anxiety/depression at baseline were included in the analyses.	**Outcome:** Anxiety/depression. **Measures:** Assessed using Child Behaviour Checklist (CBCL) completed by parents at the follow-up visit.	Multivariate mixed-effect linear regression modeling was applied to examine the effect of childhood sleep problems on anxiety/depression at adolescence, controlling for confounding factors.	Short duration of sleep (<7.5 h/night) at childhood was marginally predictive of the anxiety/depression at adolescence but not significant at 5% Type I error rate (OR = 3.3, 95% CI = 0.83–13.5).	**Strengths and Limitations:** Sleep apnea was measured objectively using polysomnograms. However, the actual measures on sleep apnea were used in the analyses. Instead, the duration of sleep was used as the outcome variable, although it would be argued that a short duration of sleep was highly correlated to the symptoms of sleep apnea. Some important confounding variables, such as parental mental health, might be missing
Armstrong et al. (2013), Wisconsin, USA	The sample was drawn from the Wisconsin Study of Family and Work for this secondary data analysis study.	Families were recruited from pregnancy and followed until the child reached 18 years. Of the original 570 families recruited, 341 had complete data on both childhood sleep problems at the age of 4.5 and 9 years and mental health at age 18 (n = 341).	**Exposure:** Sleep problems included insomnia, sleep movement, hypersomnia, and a range of parasomnias. Due to low frequencies in other problems, the study focused on persistent insomnia and sleep movement defined as having the individual problem at both 4.5 and 9 years. **Measures:** Sleep problems were assessed using the Children’s Sleep Habits Questionnaires (SHQs) by maternal reports. **Confounding variables:** Potential confounding variables were included in the final analyses. These were the child’s sex, medication of psychostimulant.	**Outcome:** Anxiety, depression, externalizing behaviors, and ADHD **Measures:** Assessed using the MacArthur Health and Behavior Questionnaire (HBQ) self-reported by the child at the age of 18.	Multivariate analysis of variance (MONOVA) was used to examine the association between persistent sleep problems at age 9 and mental health problems at 18 years controlling for confounding factors.	Persistent insomnia was significantly related to anxiety and externalizing behaviours in adolescence (F_(2,334)_ = 4.82, *p* = 0.009 and F_(2,334)_ = 4.53, *p* = 0.011). Persistent sleep movement was also significantly associated with ADHD at adolescence (F_(2,334)_ = 6.68, *p* = 0.001).	**Strengths and Limitations:** The study was well-executed and the follow-up process was complete with comparisons on many demographic variables between the participating and on-participating families. Two main limitations were identified: (1) most demographic variables were not included in the analyses to be considered as potentially confounding; (2) assessments on the exposure and outcome variables were conducted by self-reported questionnaires risking certain degree of report bias by mothers and children. (3) Other important confounding variables, such as parental mental health, might be missing.
Wang et al. (2016), Perth, Western Australia	This secondary data analysis study drew the sample from the Western Australian Pregnancy Cohort (Raine) Study.	Women between 16 and 20 weeks gestation were recruited from the public antenatal clinic at the participating sites. Mother had consented to be followed annually until the child reached the age of 18. Assessments were conducted via questionnaire and physical examination. This study focused on childhood sleep data collected at 5, 8, 10, 14-year follow-up and mental health assessment at 17 years (n = 1182).	**Exposure:** Childhood sleep problems with common symptoms of sleep disorders, including nightmares, sleepwalking and talking, trouble in falling asleep. **Measures:** The Sleep Problem Scale of the Child Behavior Checklist (CBCL). However, the focus of the study was the latent trajectory classes of sleep problems from age 5 to 14. Early childhood sleep problem was classified as normal and troubled sleepers using unconditional growth mixture models. **Confounding variables:** Not mentioned in the study.	**Outcome:** Anxiety, depression, externalizing behaviors, and ADHD **Measures:** Assessed using the Youth Self Report (YSR), which is the youth version of the self-reported by the child at the age of 17.	Simple independent sample Student’s t-tests were applied to examine the differences in the scores on the YSR between normal and troubled sleepers.	There was a significant difference in the mean score of ADHD between groups with troubled sleepers scored higher (Mnormal = 4.93, Mtroubled = 5.73, *p* < 0.05). The mean score of the troubled sleepers was also significantly higher than that of the controls (Mnormal = 7.79, Mtroubled = 9.04, *p* < 0.05). No significant differences in anxiety and depression were observed between groups.	**Strengths and Limitations:** This secondary data analysis study utilized a data set with a large sample size and a long period of follow-up. Similar to the Armstrong et al.’s study, two main limitations were identified: (1) assessments on the exposure and outcome variables were conducted by self-reported questionnaires, thus, the risk of report basis by mothers and children was high; (2) simple comparisons on main outcome variables between groups were conducted without any considerations of potential confounding factors on the results. (3) Should also consider confounding issues.
Lereya et al. (2017), Avon, UK	This was another secondary data analysis study using data collected in the Avon Longitudinal Study of the Parents and Children (ALSPAC) in the UK.	Mother and child dyads were recruited in the Avon birth cohort study with an initial 14,541 pregnant women enrolled. Parents responded to postal questionnaires on their child’s health development during follow-up. The Child was assessed annually via face-to-face interviews on psychosocial and physical health. This study utilized data collected on borderline personality disorder when the child was about 12 years (mean age 11.8 years) (n = 6050).	**Exposure:** Childhood sleep problems included nightmares, sleep maintenance problems, and trouble falling asleep. **Measures:** Sleep problems were assessed by a set of sleep questions when the child was 2.5, 3.5, 4.8, and 6.8 years of age. In this study, sleep problems were categorized in accordance to the responses at each time point for each type of problem. For example, no nightmare at all, nightmare at 1 time point, 2 time points, 3 or more time points. **Confounding variables:** A number of potential confounding variables were controlled. These included: sex, emotional temperament at 2 years, family adversity index, physical and sexual abuse at 2.5, 3.5, 4.8, or 6.8 years, preschool maladaptive parenting, Developmental and Wellbeing Assessment at 7 years, and emotional and behavioral problems at 9.5 and 11.7 years.	**Outcome:** Borderline personality disorder (BPD) symptoms. **Measures:** Assessed by a trained psychologist at face-to-face interview using the UK Childhood Interview for DSM-IV-Borderline Personality Disorder at about 12 years.	Logistic regression analysis as the primary analytical approach and verified with path analysis	Having persistent nightmares (i.e., nightmares assessed as positive at 3 or more time points) during early childhood was significantly associated with BPD symptoms at adolescence after controlling for potential confounding variables (OR = 1.62, 95% CI = 1.12–2.32). Results were also verified with path analysis.	**Strengths and Limitations:** Similar to the previous study in this review, this secondary data analysis study utilized a large data set that could provide sufficient power for demonstrating a true effect between the exposure and outcome variables. Another strength of the study was that sufficient control for potential confounding effects was in place to ensure the precision of the effect estimate.
Morales-Munoz et al. (2020), Avon, UK	This was another secondary data analysis study using data of the same Avon Longitudinal Study of the Parents and Children (ALSPAC) in UK as Lereya et al.	Mother and child dyads were recruited in the Avon birth cohort study with an initial 14,541 pregnant women enrolled. Parents responded to postal questionnaires on their child’s health development during follow-up. The Child was assessed annually via face-to-face interviews on psychosocial and physical health (n = 7155). This study utilized data collected on Psychotic symptoms and BPD since the child was 12 years. The main focus of the study was on psychotic symptoms (n = 7155).	**Exposure:** Childhood sleep problems included nightmares, duration of sleep, sleep maintenance problems, and sleep routine regularity. **Measures:** Sleep problems were assessed by a set of sleep questions when the child was 6 months, 18 months, 30 months, 3.5, 4.8, and 5.8 years of age. This study focused on the duration of sleep, sleep maintenance, and sleep routine. **Confounding variables:** A number of potential confounding variables were controlled. These included: sex, emotional temperament at 2 years, family adversity index, physical and sexual abuse, prematurity, and maternal age when the child was born.	**Outcome:** Psychotic symptoms. **Measures:** Assessed by a trained psychologist at face-to-face interview using the Psychotic-Like Symptom Interview.	Logistic regression analysis as the primary analytical approach. Of interest to the authors was also the possible mediating effect of depression at 10 years on the relationship between early childhood sleep problems and psychotic symptoms with path analysis which was similar to Lereya’s study.	There were significant relationships between a regular sleep routine and psychotic symptoms. Children with a regular sleep routine at 6 months had a reduced odds of psychotic symptoms at 12 to 13 years by about 30% (OR = 0.68, 95% CI = 0.50–0.93). Children with a regular sleep routine at 30 months had a reduced odds of psychotic symptoms of about 35% 4 (OR = 0.68, 95% CI = 0.44–0.95). Children with a regular sleep routine at 5.8 years had an even greater reduction in odds of psychotic symptoms at 12 to 13 years by about 70% (OR = 0.32, 95% CI = 0.19–0.53). Results were obtained after the adjustment of confounding variables. A possible mediating effect of depression on children’s regular sleep routine at 5.8 years and psychotic symptoms at 12 to 13 years was also reported.	**Strengths and Limitations:** Similar to Lereya’s study utilizing the same data set, this study shared the same strengths that the large sample size could provide sufficient power for demonstrating a true effect between the exposure and outcome variables. Similarly, sufficient control for potential confounding effect was in place to ensure the precision of the effect estimate was another strength of the study.
Slykerman et al. (2020), Auckland, New Zealand	This was a secondary data analysis study utilizing data collected from the Auckland Birthweight Collaborative Study (ABC Study).	Mothers, mainly of European ethnicity, and their newborn children were recruited to the Study. All children were born full-term with a gestation of 37 weeks or longer between October 1995 and November 1997. Children were then followed until 11 years. Data on sleep problems were collected at 7 years and mental health problems were assessed at 11 years. In total, 547 children had provided data on both the exposure and outcome variables. (n = 547).	**Exposure:** Sleep duration at 7 years was the only sleep variable of interest. **Measures:** Sleep duration was measured by actigraphy for 24 h. **Confounding variables:** Birthweight, sex, gestation, socioeconomic status, maternal smoking during pregnancy, marital status at the time the child was born, maternal school leaving age, and child intelligence were included in the analyses for adjustment.	**Outcome:** Emotional/behavioral difficulties and ADHD. **Measures:** Emotional/ behavioral difficulties were assessed by the Strengths and Difficulties Questionnaire (SDQ) self-reported by both parent and child at age 11. For ADHD, it was assessed using the Conner’s Rating Scale (CRS) filled in by parents and teachers when the child was 11 years old.	Logistic regression analyses were applied to examine the relationship between the exposure and outcome variables.	After adjusting for potential confounding factors, there was no significant relationship between sleep duration at 7 years and emotional/behavioral problems as well as ADHD at 11 years.	**Strengths and Limitations:** Similar to other studies in this review, this study suffered similar shortcomings as the other. Despite the objective measures of sleep problems using actigraphy, sleep duration was the only variable under investigation. Given the sound approach in exposure assessment, other sleep variables should also be considered. The self-reporting on the outcome variables might incur some report bases by parents, children, and teachers. Another shortcoming of this study was the lack of information on sleep problems of children at their earlier stage of development.

**Table 2:** Summaries on the relationships between early childhood sleep problems and adolescent mental health resulting from the review of the selected studies.

				Studies			
	Gregory and O’Connor	Silva et al.	Armstrong et al.	Wang et al.	Lereya et al.	Morales-Munoz et al.	Slykerman et al.
Sleep Problems	Common Symptoms of Sleep Disorders	Short Duration of Sleep (<7.5 h/Night)	Persistent Insomnia	Common Symptoms of Sleep Disorders	Persistent Nightmares	Irregular Sleep Routine	Sleep Duration
Mental Health Problems							
Anxiety/Depression	+	−	+				
ADHD	+		+	+			
Externalizing behaviors			+	+			
BPD					+		
Psychotic symptoms						+	
Emotional/Behaviral difficulties							−
